# Spin transport in undoped InGaAs/AlGaAs multiple quantum well studied via spin photocurrent excited by circularly polarized light

**DOI:** 10.1186/s11671-015-1218-3

**Published:** 2016-01-07

**Authors:** Laipan Zhu, Yu Liu, Wei Huang, Xudong Qin, Yuan Li, Qing Wu, Yonghai Chen

**Affiliations:** Key Laboratory of Semiconductor Materials Science, Institute of Semiconductors, Chinese Academy of Sciences, Beijing, 100083 People’s Republic of China; Beijing Institute of Nanoenergy and Nanosystems, Chinese Academy of Sciences, National Center for Nanoscience and Technology (NCNST), Beijing, 100083 People’s Republic of China

**Keywords:** Spin diffusion, Spin drift, The circularly polarized light, The reciprocal spin Hall effect

## Abstract

The spin diffusion and drift at different excitation wavelengths and different temperatures have been studied in undoped InGaAs/AlGaAs multiple quantum well (MQW). The spin polarization was created by optical spin orientation using circularly polarized light, and the reciprocal spin Hall effect was employed to measure the spin polarization current. We measured the ratio of the spin diffusion coefficient to the mobility of spin-polarized carriers. From the wavelength dependence of the ratio, we found that the spin diffusion and drift of holes became as important as electrons in this undoped MQW, and the ratio for light holes was much smaller than that for heavy holes at room temperature. From the temperature dependence of the ratio, the correction factors for the common Einstein relationship for spin-polarized electrons and heavy holes were firstly obtained to be 93 and 286, respectively.

## Background

Much attention has been given to semiconductor spintronics for the promising applications in information technology [1, 2]. One of the fundamental issues on semiconductor spintronics is the spin transport and its manipulation, including spin-related diffusion and drift. The anomalous circular photogalvanic effect (ACPGE) [3–5] and anomalous Hall effect (AHE) [6–10], which are derived from the same spin-orbit coupling (SOC) mechanisms (intrinsic or extrinsic) based on the reciprocal spin Hall effect (RSHE), open avenues to the study of the relationship between the diffusion and the drift of photoinduced spin-polarized electrons. According to [11], the ratio of the diffusion coefficient to the mobility of the photoinduced spin-polarized electrons has been measured to be 0.08 V in AlGaN/GaN heterostructure with the excitation wavelength of 1064 nm at room temperature. In this work, we focused on the spectrum and temperature dependence of the transport properties corresponding to interband transitions in an undoped InGaAs/AlGaAs multiple quantum well (MQW) in which a strong Rashba SOC had been demonstrated in previous studies [10, 12, 13].

**Fig. 1:**
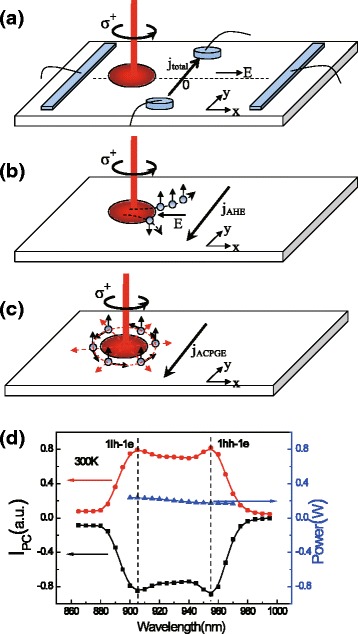
The schematic diagram of the total spin-related current, AHE and ACPGE, and the spectra of common photoinduced currents. **a** The schematic diagram of the geometry of the experiment. The external electric field is applied via the strip electrodes. The total spin-related current is measured by the two circle electrodes. **b** A schematic diagram of the AHE. **c** A schematic diagram of the ACPGE. **d** The spectra of common photoinduced currents on the circle electrodes at 300 K. The *red circle dots* and *black square dots* denote the currents under reversed electric fields. The *blue triangle dots* denote the power corresponding to each wavelength

The intensity of the circularly polarized light has a Gauss profile, thus an inhomogeneous spin density will be excited on the sample plane. One can assume that the effective spin density (*N*_eff_) flowing through the region of *x*=0 has a Gaussian distribution [11, 13], i.e., $N_{\text {eff}}=g\tau _{s}\frac {c}{\sigma }e^{-x^{2}/\sigma ^{2}}$, where *g*,*τ*_*s*_,*c*, *x*, and *σ* are the generation rate of spin-polarized carriers, the spin relaxation time, an arbitrary constant, the spot coordinate along the *x* axis, and the standard deviation of the Gaussian distribution, respectively. On the one hand, under normal incidence, the gradient of the spin density will induce a diffused spin polarization current (SPC): $\textit {\textbf {q}}_{r}^{z}=-{D_{s}}\nabla _{r}\textit {n}^{z}(\textit {r})$, where *D*_*s*_ is the spin diffusion coefficient of the photoinduced carriers, *n*^*z*^(*r*) the spin density along the *z* direction, and *r* the radial direction in the *x*−*y* plane. According to the RSHE [3, 14], a transverse electric current (density) perpendicular to both the direction of the SPC and the direction of the spin polarization is produced, which can be expressed as $\textit {j}=\gamma {e}\textit {\textbf {q}}_{r}^{z}\times \hat {\textit {z}}$, where *e*, *γ* are the elementary charge, the spin-orbit interaction coefficient based on RSHE, respectively. As a result, a swirling electric current will be induced around the light spot, which will further generate an observed ACPGE current *j*_ACPGE_/*e*=−*γD*_*s*_∇*N*_eff_ (shown in Fig. 1c). On the other hand, when a circularly polarized light irradiates vertically on the sample, the flow of the spin-polarized carriers driven by the longitudinal electric field will also lead to a transverse AHE current which is also derived from the RSHE, as shown in Fig. 1b [6–10]. And the AHE current can be expressed as *j*_AHE_/*e* = *γμ*_*s*_*EN*_eff_ [11], where *μ*_*s*_ is the spin mobility of the photoinduced carriers, and *E* is the external electric field. Thus, the total spin-related photoinduced current along the two circle electrodes can be expressed as

(1) $$ {j_{\text{total}}}/e~=~\gamma\tau_{s}\frac{gc}{\sigma}e^{-x^{2}/\sigma^{2}}\left(\frac{2D_{s}}{\sigma^{2}}x+E\mu_{s}\right),  $$

where *j*_total_ is equal to *j*_ACPGE+AHE_.

## Methods

The sample studied here is an undoped In _0.15_*Ga*_0.85_ As/Al _0.3_*Ga*_0.7_As MQW grown by molecular beam epitaxy. A 200-nm buffer layer is initially deposited on (001) SI-GaAs substrate, followed by ten periods of 100-Å In _0.15_*Ga*_0.85_As/ 100-Å Al _0.3_*Ga*_0.7_As QWs. Then, a 500 Å Al _0.3_*Ga*_0.7_As layer and 100 Å GaAs cap layer are deposited. The sample is cleaved into a narrow strip along the GaAs [1$\bar {1}$0] direction with a width of 4 mm and a length of 12 mm. The geometry has been shown in Fig. 1a, where two circle ohmic electrodes (whose radius are both 0.25 mm) with a distance of 2.5 mm and two strip ohmic electrodes (whose size are both 0.5 × 3 mm) with a distance of 10 mm were made along *y* and *x* direction, respectively, by indium deposition and annealed at about 420 °C in nitrogen atmosphere.

The experimental setup is described as follows. A modelocked Ti:sapphire laser with a repetition rate of 80 MHz serves as the radiation source. The incident light goes through a polarizer and a photoelastic modulator (PEM), of which the peak retardation is set to be *λ*/4, to yield a modulated circularly polarized light with a fixed modulating frequency at 50 KHz. By using an optical chopper with the rotation frequency of 223 Hz, the spectra of common photoinduced currents (*I*_PC_) are also measured for comparison, which show clearly the energy positions corresponding to 1hh-1e (the first valence subband of heavy holes to the first conduction subband) and 1lh-1e (the first valence subband of light holes to the first conduction subband) transitions (see Fig. 1d). The Gaussian profile light beam irradiates vertically on the sample with a diameter of about 1.7 mm at the perpendicular bisector of the two circle electrodes. The external electric field applies to the strip electrodes. The photogalvanic currents are collected through the two circle electrodes by two lock-in amplifiers with the synchronization frequencies set to be 50 KHz and 223 Hz, respectively.

## Results and discussion

**Fig. 2:**
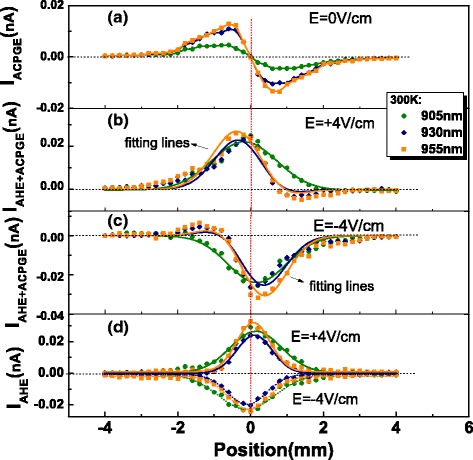
The transverse spin-related currents at room temperature and different wavelengths corresponding to different longitudinal electric fields. The transverse spin-related currents at different wavelengths corresponding to longitudinal electric fields of **a** 0 V/cm, **b** + 4 V/cm, and **c** −4 V/cm as a function of light spot position. All *lines* in **a** are for ease of viewing, and *lines* in **b** and **c** are curves fitted in Eq. 1. **d** The AHE currents extracted by subtracting the ACPGE currents in **a** from the total spin-related photocurrents in **b** and **c**. The *solid* and *dotted lines* are curves of Gaussian fitting corresponding to AHE

In the experiment, the total spin-related currents at electric fields of 0, + 4, and −4 V/cm are measured as a function of the spot location at three different wavelengths. As shown in Fig. 2a, under the electric field of 0 V/cm, the total spin-related currents which are actually contributed only from ACPGE currents reverse the sign from the left to right side. The electric fields selected to be + 4 and −4 V/cm ensured that the AHE currents were comparable with the ACPGE currents. The total spin-related currents were fitted very well in Eq. 1 (shown in Fig. 2b, c). The AHE current was extracted by subtracting the ACPGE current from the total spin-related photocurrent. The extracted curves in Fig. 2d exhibit symmetric Gaussian-like distribution, and the directions corresponding to electric fields of + 4 and −4 V/cm are opposite, being consistent with the mechanism of AHE.

**Fig. 3:**
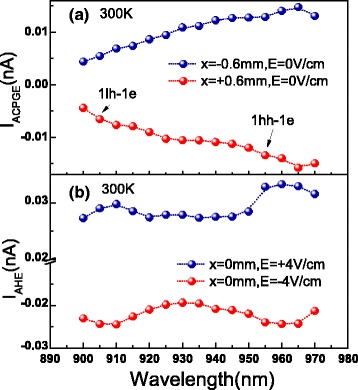
ACPGE and AHE currents corresponding to the extreme points as a function of wavelength, respectively. **a** ACPGE currents corresponding to the extreme points (*x*=±0.6 mm) as a function of wavelength. **b** AHE currents corresponding to the extreme points (*x*=0 mm) as a function of wavelength. The *blue* and *red dotted lines* are for ease of viewing

Next, we extracted the extreme values of ACPGE and AHE currents from Fig. 2a, d, respectively. To our surprise, the extracted curve shape in Fig. 3a is quite different from that in Fig. 3b. In concrete terms, there are no obvious peaks corresponding to 1hh-1e and 1lh-1e in Fig. 3a, while there are distinct peaks corresponding to 1hh-1e and 1lh-1e in Fig. 3b, which are due to that the diffusion coefficient and the mobility of the photoinduced spin-polarized carriers have different relationships with excitation wavelength. According to [11], by observing the two extreme points of total spin-related current, the ratio of the spin diffusion coefficient to the mobility of the photoinduced spin-polarized electrons can be calculated directly. However, in our experiment, the extreme positions are not always clear to be figured out especially at small wavelengths (see Fig. 2b, c). Despite this, one can still get the ratio of the spin diffusion coefficient to the mobility of the photoinduced spin-polarized carriers by fitting the experiment data using Eq. 1.

**Fig. 4:**
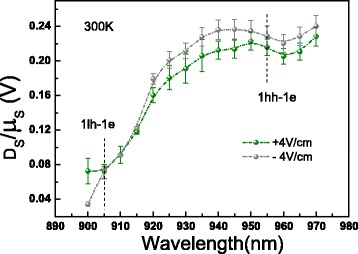
The ratio of the spin diffusion coefficient to the mobility of the photoinduced spin-polarized carriers as a function of wavelength at 300 K. The ratio of the spin diffusion coefficient to the mobility of the photoinduced spin-polarized carriers as a function of wavelength at 300 K

**Fig. 5:**
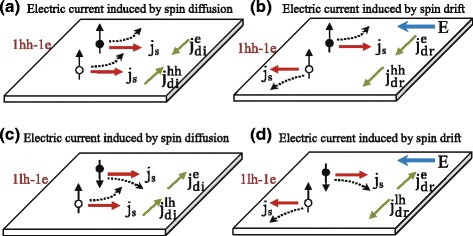
The schematic diagrams of electric current induced by spin diffusion and spin drift. **a** and **c** are schematic diagrams of electric current induced by spin diffusion corresponding to 1hh-1e and 1lh-1e, respectively. **b** and **d** are schematic diagrams of electric current induced by spin drift corresponding to 1hh-1e and 1lh-1e, respectively. In these four figures, the *bigger black* (*white*) *dots* with a *black arrow* denote spin-polarized electrons (holes), the *red arrows* denote the spin currents, the *blue arrows* denote the electric fields, and the *green arrows* denote the electric currents

From the fitted data in Fig. 4, we can see that the ratio is independent of external electric field, and the ratio remains almost a constant around 1hh-1e while it decreases sharply when decreasing the wavelength to 1lh-1e. In order to understand the possible reasons, we have to take the spin current from holes into consideration. According to the experiments of ISHE from [6, 15], we can deduce that the directions of the spin transverse force for electrons and holes are the same if the directions of spin polarization are the same and the directions of spin current are also the same; as a result, the directions of the spin-polarized electric currents for electrons and holes are opposite. Therefore, we can depict the detailed spin diffusion electric currents and spin drifting electric currents corresponding to 1hh-1e and 1lh-1e, respectively (see Fig. 5). For 1hh-1e transition, the effective spin density can be simplified as *N*_eff_=*τ*_*e*_*N*_0_ for electrons and *N*_eff_=*τ*_*hh*_*N*_0_ for heavy holes, where $N_{0}=g_{0}\frac {c}{\sigma }e^{-x^{2}/\sigma ^{2}}$ is a constant (where *g*_0_ is the generation rate for 1hh-1e transition), *τ*_*e*_ is the spin relaxation time of electrons, and *τ*_*hh*_ is the spin relaxation time of heavy holes. For 1lh-1e transition, *N*_*eff*_ can be simplified as *N*_eff_=*τ*_*e*_*N*_1_ for electrons and *N*_eff_=*τ*_*lh*_*N*_1_ for light holes, where $N_{1}=g_{1}\frac {c}{\sigma }e^{-x^{2}/\sigma ^{2}}$ is a constant (where *g*_1_ is the generation rate for 1lh-1e transition), and *τ*_*lh*_ is the spin relaxation time of light holes. So, the total spin-polarized electric current corresponding to 1hh-1e and 1lh-1e can be expressed as

(2) $$ \begin{aligned} {j_{1hh-1e}}~=~&(D_{e}\gamma_{e}\tau_{e}-D_{hh}\gamma_{hh}\tau_{hh})e\nabla{N_{0}}\\ &+(\mu_{e}\gamma_{e}\tau_{e}+\mu_{hh}\gamma_{hh}\tau_{hh})eE{N_{0}} \end{aligned}  $$

and

(3) $$ \begin{aligned} {j_{1lh-1e}}~=~&(-D_{e}\gamma_{e}\tau_{e}-D_{lh}\gamma_{lh}\tau_{lh})e\nabla{N_{1}}\\ & +(-\mu_{e}\gamma_{e}\tau_{e}+\mu_{lh}\gamma_{lh}\tau_{lh})eE{N_{1}}, \end{aligned}  $$

respectively.

Thus, the ratios of the spin diffusion coefficient to the mobility of the photoinduced spin-polarized carriers can be defined as

(4) $$ \left(\frac{D_{s}}{\mu_{s}}\right)_{1hh-1e}~=~\frac{D_{e}\gamma_{e}\tau_{e}-D_{hh}\gamma_{hh}\tau_{hh}}{\mu_{e}\gamma_{e}\tau_{e}+\mu_{hh}\gamma_{hh}\tau_{hh}}  $$

for 1hh-1e transition and

(5) $$ \left(\frac{D_{s}}{\mu_{s}}\right)_{1lh-1e}~=~\frac{D_{e}\gamma_{e}\tau_{e}+D_{lh}\gamma_{lh}\tau_{lh}}{\mu_{lh}\gamma_{lh}\tau_{lh}-\mu_{e}\gamma_{e}\tau_{e}}  $$

for 1lh-1e transition. The Rashba effect which is stronger for the first valence band than that for the first conduction band and is stronger for the first light hole subband than that for the first heavy hole subband in *p*-type quantum wells has been demonstrated by several authors [16–18]. Supposing the situation for undoped MQW is similar to that for the *p*-type quantum wells. Thus, the reciprocal spin Hall coefficient (which is proportional to Rashba effect) of light holes (*γ*_*lh*_) is probably far larger than that of electrons (*γ*_*e*_). Assuming *μ*_*lh*_ is comparable with *μ*_*e*_, while *D*_*lh*_ is far smaller than *D*_*e*_, and *τ*_*e*_ is at the same order with *τ*_*lh*_, only then can we get *μ*_*lh*_*γ*_*lh*_*τ*_*lh*_≫*μ*_*e*_*γ*_*e*_*τ*_*e*_. As a result, $\left (\frac {D_{s}}{\mu _{s}}\right)_{1lh-1e}$ is smaller than $\left (\frac {D_{s}}{\mu _{s}}\right)_{1hh-1e}$ (see Fig. 4). Around the 1hh-1e transition, the contribution to the ratio of the spin diffusion coefficient to the mobility of spin-polarized carriers is mainly from the 1hh-1e transition. With wavelength further decreasing, the contribution to the ratio from 1hh-1e transition became more and more weak, while the contribution from 1lh-1e became dominant. Therefore, the ratio remains almost a constant around 1hh-1e while it decreases sharply when decreasing the wavelength to 1lh-1e.

**Fig. 6:**
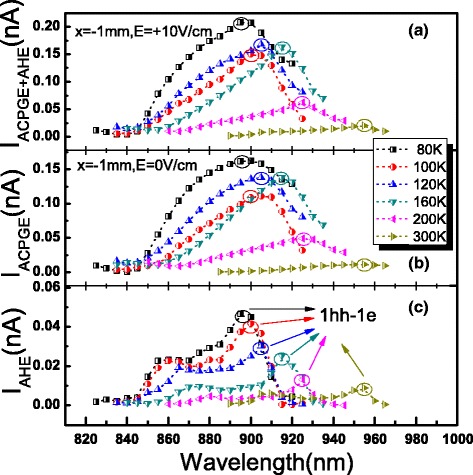
The spectra of total spin-related photoinduced currents, ACPGE currents and AHE currents, at different temperatures. **a** The spectra of total spin-related photoinduced currents at different temperatures. The spot location is at *x*=−1 mm, and the external electric field is + 10 V/cm. **b** The spectra of ACPGE currents at different temperatures. The spot location is at *x*=−1 mm, and the external electric field is 0 V/cm. **c** The spectra of AHE currents at different temperatures which are obtained by subtracting the data of **b** from the corresponding data of **a**

**Fig. 7:**
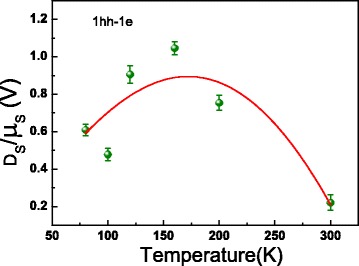
The ratio of the spin diffusion coefficient to the mobility of the photoinduced spin-polarized carriers as a function of lattice temperature corresponding to 1hh-1e transition. The ratio of the spin diffusion coefficient to the mobility of the photoinduced spin-polarized carriers as a function of lattice temperature corresponding to the 1hh-1e transition. The *solid line* is the fitting curve using Eq. 6

At last, we studied the temperature dependence of ACPGE and AHE currents. From Fig. 6, we can see that the ACPGE and AHE currents are significantly enhanced with temperature decreasing, especially at 1hh-1e transition. The spectra of AHE currents at different temperatures in Fig. 6c are obtained by subtracting the data in Fig. 6b from the corresponding data in Fig. 6a. If we just consider the 1hh-1e transition, by contrasting the value of ACPGE current with that of AHE current at different temperatures, we can further deduce the temperature dependence of $\frac {D_{s}}{\mu _{s}}$. As shown in Fig. 7, we have found that the ratio first increased and then decreased when the temperature decreased from room temperature. In these quantum wells, electron spin relaxation time is considered to vary approximately as *T*^−2^, i.e., *τ*_*e*_=*A*_*e*_*T*^−2^, where *A*_*e*_ is a constant, which is quiet different from the assumption of a narrow quantum wells in [2, 10, 19]; and the hole spin relaxation time is roughly proportional to *T*^−1^, i.e., *τ*_*hh*_=*A*_*hh*_*T*^−1^, where *A*_*hh*_ is a constant [20]. Assuming that $\frac {D_{e}}{\mu _{e}}=\frac {\chi _{e}{k_{B}{T}}}{e}$ and $\frac {D_{\textit {hh}}}{\mu _{\textit {hh}}}=\frac {\chi _{\textit {hh}}{k_{B}{T}}}{e}$, where *χ*_*e*_ and *χ*_*hh*_ are correction factors for the common Einstein relationship for the spin-polarized electrons and holes, respectively. At high temperatures (≥80 K), the mobility of GaAs/AlGaAs two-dimensional electron and hole gases are respectively proportional to *T*^−2.4^ and *T*^−2^ given by former studies [21, 22]. For spin-polarized electrons and holes, in order to simplify the discussion, we suppose the ratio of spin mobility between spin-polarized electrons and spin-polarized holes is proportional to *T* (there were few relevant reports on the temperature dependence of spin mobility), i.e., $\frac {\mu _{e}}{\mu _{\textit {hh}}}=\lambda _{0}T$, where *λ*_0_ is a constant. Then, Eq. 4 can be further expressed as

(6) $$ \left(\frac{D_{s}}{\mu_{s}}\right)_{1hh-1e}~=~\frac{(\chi_{e}k_{B}{A_{e}}\gamma_{e})T-(\chi_{hh}k_{B}{A_{hh}}\gamma_{hh})T^{3}}{(e{A_{e}}\gamma_{e})+(e{A_{hh}}\gamma_{hh})T^{2}}.  $$

Ignoring the temperature dependence of *γ*_*e*_ and *γ*_*hh*_, one can use Eq. 6 to fit the data in Fig. 7; and the correction factors for Einstein relationship for spin-polarized electrons and heavy holes are fitted as *χ*_*e*_=93 and *χ*_*hh*_=286, respectively. The factor of spin-polarized heavy holes is almost 3 times larger than that of spin-polarized electrons. According to the Einstein relationship for electron transport, the value of $\frac {D}{\mu }$ is estimated to be about 0.026 V at room temperature, which is much smaller than the value of $\frac {D_{s}}{\mu _{s}}$ we obtained in this work. We would like to clarify that $\frac {D_{s}}{\mu _{s}}$ for spin transport is not necessarily the same as $\frac {D}{\mu }$ for electron transport. The essential difference is that the Einstein relationship is derived based on the conservation law of electrons; however, spin is not conservative [11]. We believe that the Einstein relationship for spin should be different for different semiconductor materials.

## Conclusions

In conclusion, the spin diffusion and drift at different wavelengths and different temperatures have been studied in undoped InGaAs/AlGaAs MQW. By using the AHE and ACPGE which are all derived from RSHE, we obtained the ratio between the spin diffusion coefficient and the mobility of spin-polarized carriers. From the wavelength dependence of the ratio, we found that the spin diffusion and drift of holes became as important as electrons in this undoped MQW, and the ratio for light holes was much smaller than that for heavy holes at room temperature. From the temperature dependence of the ratio corresponding to the 1hh-1e transition, we believed the ratio is contributed by the combined effect of spin-polarized electrons and spin-polarized heavy holes. The correction factors for the common Einstein relationship for spin-polarized electrons and heavy holes are firstly obtained to be 93 and 286, respectively. It is worth noting that the AHE and ACPGE measurements used in this study are conducted under ambient conditions with a simple setup and operation, which provides a good method for the study of spin-related diffusion and drift.
